# Scaling up frailty: psychometric validation of the functional limitations and geriatric syndromes frailty questionnaire—a new tool for uniformly classifying vulnerable hospital patients

**DOI:** 10.3389/fmed.2025.1642562

**Published:** 2025-10-03

**Authors:** Bruno Bernardini, Rosa Pedale, Paola Arosio, Federico Piccioni, Stefano Mancin, Francesco Reggiani, Giovanna Cerina, Sara Ghirmai, Carla Corsini, Riccardo Levi, Maria Vittoria Fantacci, Laura Tabarretti, Giulia Goretti, Rodolfo Hurle

**Affiliations:** ^1^Neuro-Center, Neuro-Rehabilitation Unit, Department of Rehabilitation, IRCCS Humanitas Research Hospital, Milan, Italy; ^2^Internal Medicine-Nephrology and Dialysis Unit, Department of Internal Medicine, IRCCS Humanitas Research Hospital, Milan, Italy; ^3^Hospitalist Service, Department of Internal Medicine, IRCCS Humanitas Research Hospital, Milan, Italy; ^4^Anesthesia Unit 1, Department of Anesthesia and Intensive Care, IRCCS Humanitas Research Hospital, Milan, Italy; ^5^Cancer-Center, Clinical Nutrition Unit, IRCCS Humanitas Research Hospital, Milan, Italy; ^6^Department of Biomedical Sciences, Humanitas University, Milan, Italy; ^7^Department of Neuroradiology, IRCCS Humanitas Research Hospital, Milan, Italy; ^8^Department of Urology, IRCCS Humanitas Research Hospital, Milan, Italy; ^9^Physiotherapy Service, Neuro-Rehabilitation Unit, Department of Rehabilitation, IRCCS Humanitas Research Hospital, Milan, Italy; ^10^Department of Quality Management, IRCCS Humanitas Research Hospital, Milan, Italy

**Keywords:** frailty, psychometric, confirmatory factor analysis, item response theory, integrated care pathways, patient care planning

## Abstract

**Background:**

Comprehensive psychometric validation is essential to obtain a common metric for reliable diagnostic and prognostic decision-making in frailty. In this study, we used a single-factor approach to derive and psychometrically validate a standardized frailty measure from 23 reflective items (eight functional limitations and 15 geriatric syndromes) from a new, multidomain, questionnaire. We used confirmatory factor analysis (CFA) and item response theory (IRT) to achieve this goal.

**Methods:**

This single-centre, cross-sectional study included a convenience sample of 900 community-dwelling patients (median age: 73.4 years; IQR: 67.0–81.6; 59.7% male) undergoing elective surgery (*n =* 568, 63.1%) or admitted to the internal medicine unit for acute illnesses (*n =* 332, 36.9%). Of the elective patients, 50.4% completed the questionnaire via a web platform. The rest completed the questionnaire during a face-to-face interview at their preoperative visit or within 48 h of admission.

**Results:**

The CFA validated the single-factor solution for 16 of the 23 items in the questionnaire and confirmed the good internal consistency of the construct. IRT analyses showed that the 16 items of the Functional Limitation and Geriatric Syndrome Frailty Questionnaire (FLIGS-FQ-16) have good discriminatory power, satisfactory threshold parameters, and equal function for men and women. The FLIGS-FQ-16 total score provides reliable information on the severity of frailty, ranging from 0.18 standard deviations below the population mean (“not frail”) to 2.7 standard deviations above the population mean (“severely frail”). Applying the standardized FLIGS-FQ-16 threshold scores to our sample, we found an overall prevalence of frailty of 40.9%, with a significant difference between acute patients (75.3%) and elective patients (20.8%, *p* < 0.001). Among acute patients, 37.6% were moderately or severely frail. Among elective patients, 19.0% were moderately frail and 1.8% were severely frail.

**Conclusion:**

The five functional limitations and 11 geriatric syndromes of the FLIGS-FQ-16 aggregate into a robust single-factor construct with adequate psychometric properties that uniformly measure frailty up to the most severe levels. In addition to serving as a screening tool, the FLIGS-FQ-16 is useful for making individualized decisions and developing personalized treatment plans in perioperative medicine and the management of hospitalised older adults because it is based on treatable risk factors.

## 1 Background

As the population ages, a growing number of hospital patients are older adults with multiple chronic conditions and disabilities, including frail individuals (1). Therefore, identifying the most vulnerable patients early on presents a major challenge to clinicians and health policymakers when designing safe and effective clinical pathways.

Frailty is an age-related, multi-systemic syndrome that reduces an individual’s ability to cope with stress by decreasing their homeostatic reserves (2). Frailty is a marker of vulnerability. A substantial body of literature spanning virtually all medical and surgical specialties has established a link between frailty and an increased likelihood of adverse health outcomes, including morbidity, mortality, functional decline and institutionalisation.

Despite this evidence, two significant gaps in our understanding persist, calling into question the diagnostic and prognostic accuracy of frailty measures and, consequently, their reliability in informing individual patient decision-making (3–6).

Firstly, although frailty is an unobservable latent trait, most instruments used to measure it have only undergone clinimetric validation. This bypasses the preliminary psychometric validation that is fundamental to ensuring construct reliability and appropriate item weighting (7–10). In fact, overlapping domains or duplicate items within the same instrument render the construct unreliable (11), resulting in inflated and overly optimistic frailty measures that cannot be compared or generalised. Of the instruments most frequently used to measure frailty, only the Tilburg Frailty Indicator (12) and the Groeningen Frailty Indicator (13) have undergone adequate validation in terms of construct dimensionality and consistency. Inadequate weighting of individual items can also lead to inaccurate frailty measurements for diagnosis, prognosis and care planning at individual level. For example, both the Fried’s frailty phenotype (14) and the Frailty Index (15) fail to prioritise issues that should be considered in treatment because they assign equal weight to all items.

In a hospital setting, all of these distortions can result in hasty decisions and inequitable care for older adults.

Secondly, as many frailty assessment tools are unable to pinpoint patients’ care needs, a Comprehensive Geriatric Assessment (CGA) is necessary to plan and implement personalized interventions. While the CGA approach is widely regarded as the most effective, inconsistent results are often observed (16, 17) due to variations in content and practices, which depend on the composition of the geriatric team and organizational context. The lack of a standardized operational approach to CGAs hinders the effective comparison of interventions for patients with different levels of frailty.

This paper introduces the Functional Limitations and Geriatric Syndromes Frailty Questionnaire (FLIGS-FQ), a new, multidomain frailty assessment tool designed to address these issues. Starting from an initial pool of items reflective of frailty, this study aims to derive and validate the FLIGS-FQ according to contemporary psychometric standards using a large sample of patients referred to our hospital for elective surgery or acute medical conditions.

## 2 Methods

### 2.1 Some basics of frailty psychometrics

As with all latent variables, frailty can be inferred and measured using observable indicators (items) that either reflect or cause it. The first step in defining a measurement model for a latent variable is to distinguishing between reflective-effect and formative-causal items (18).

According to the reflective model, exemplified by Fried’s frailty phenotype (14), frailty is understood as an endogenous process that gives rise to observable items stemming from a single underlying latent trait. All reflective items are positively intercorrelated and interchangeable, consequently, the presence or absence of a reflective item does not affect the structure or extent of the latent trait.

Conversely, the formative model, exemplified by the Frailty Index (15), posits that frailty is an exogenous process resulting from the accumulation of multiple causal items. The causal items invariably give rise to a multidimensional structure and are not interchangeable. Consequently, it is essential to carefully weigh the magnitude of each formative item, as its presence or absence can alter the structure and extent of the latent trait (19, 20).

As formative models are criticized for having an unclear theoretical basis and being difficult to interpret, single-factor reflective models are favored to create simple, psychometrically valid tools for clinical practice (21, 22).

Confirmatory Factor Analysis (CFA) and Item Response Theory (IRT) are currently the reference standards for psychometrically validating instruments that measure latent variables (23, 24).

CFA uses structural equation modelling to confirm consistency between a known or theorised latent dimensional structure and a set of observable items. Additionally, IRT probabilistically estimates the interaction between the latent factor and individual items in order to produce an unconditional measure of a person’s latent trait (25).

IRT has at least three advantages over the classical test theory, against which most frailty assessment tools currently in use have been validated.

- The IRT generates true interval scale of the extent of the latent variable, known as a theta-score. The theta score serves as a common metric for estimating item properties and latent trait levels of individuals (26). Similar to the z-score, the theta score has a mean of 0, which corresponds to the average level of the latent trait in the population, and a range that conventionally extends from −3 to +3 standard deviations from the mean. In the context of frailty, this range is interpreted as a continuum that extends from “highly robust” to “highly frail.”
- The IRT is a useful tool for constructing and selecting items that cover the relevant theta score range for the target population (27). In the context of a hospital frailty screening tool, for example, it is sufficient to prepare items that measure the severity spectrum from “non-frail” to “severely frail,” rather than measuring all levels of robustness.
- The estimates obtained from well-conducted IRT are independent of the characteristics of the sample used to develop the test (23). This property, known as parameter invariance, allows the IRT model to be generalized and applied across different populations or groups.

### 2.2 Concept and development of the FLIGS-FQ

As one of our aims was to create a questionnaire consisting entirely of items reflecting frailty, specific multimorbidity and social frailty were excluded from the FLIGS-FQ structure as formative items. Thus, our hypothesis was that functional limitations and geriatric syndromes alone could adequately represent the single construct of frailty, as they are manifestations of common underlying pathophysiological processes (28).

The FLIGS-FQ was designed as a multi-domain questionnaire to identify risk factors for frailty in patients in the month prior to their encounter with the healthcare system. We anticipated that the risk factor profile would match the medical and functional needs of patients to quantify their individual risk of adverse outcomes. We have already successfully used this approach in a prognostic study in the field of rehabilitation (29).

#### 2.2.1 Targeting needs and question-item generation

Functional limitations were framed in terms of instrumental (30) and basic (31) activities of daily living, and were selected as target conditions by consensus based on literature and their perceived importance as risk factors for adverse health outcomes during hospitalisation. All geriatric syndromes were considered target conditions for question generation as they are already recognised risk factors for frailty and adverse health outcomes.

As another aim of the FLIGS-FQ was to stratify vulnerable patients without exploring the full frailty spectrum, each question was worded to capture each target condition at a problematic threshold. Questions requiring a judgement from the respondent were labelled with an anchor (e.g., hearing aids, mobility aids or urinary incontinence pads) or terms such as “help” for functional limitations, “often” or “serious” for geriatric syndromes.

All FLIGS-FQ questions are phrased in familiar language, have the same directional wording and require a true/false response. The final pool to be submitted for psychometric validation consisted of 23 items-questions: eight for functional limitations and 15 for geriatric syndromes. Supplementary Table 1 shows how the items are arranged by domain of interest. Table 1 of the results presents the initial pool of 23 FLIGS-FQ questions.

**Table 1:** Initial pool of FLIGS-FQ items and response endorsement frequencies by patient group.

Items		Short label	Total *n =* 900	Elective surgical patients *n =* 568	Acute medical patients *n =* 332
Functional limitations					
qF1	Is supported by a caregiver or relative for more than 6 h a day	Caregiver	178 (19.8)	43 (7.6)	135 (40.7)
qF2	Must be followed or helped to bathe or shower	Bathing	203 (22.6)	40 (7.0)	163 (49.1)
qF3	Needs help getting dressed	Dressing	161 (17.9)	49 (8.6)	112 (33.7)
qF4	Needs supervision or help getting around the home	Home mobility	128 (14.2)	26 (4.6)	102 (30.7)
qF5	Needs help to manage medications	Medications	167 (18.6)	27 (4.7)	140 (42.2)
qF6	Needs frequent supervision in usual activities	Supervision	128 (14.2)	19 (3.4)	109 (32.8)
qF7	Must always be accompanied when it is necessary to leave the house	Accompaniment	281 (31.2)	90 (15.8)	191 (57.5)
qF8	Must use a cane or other aids to walk or move around outside the home	Mobility aids	261 (29.0)	84 (14.8)	177 (53.3)
Geriatric syndromes					
qS1	Has frequent dizziness or balance problems	Imbalance	198 (22.0)	45 (7.9)	153 (46.1)
qS2	Has serious vision problems	Poor vision	103 (11.4)	31 (5.5)	72 (21.7)
qS3	Has serious hearing problems or use a hearing aid	Poor hearing	121 (13.4)	44 (7.7)	77 (23.2)
qS4	Takes 5 or more medications per day (excluding supplements and vitamins)	Polypharmacy	415 (46.1)	199 (35.0)	216 (65.1)
qS5	Has major memory problems	Amnesia	126 (14.0)	18 (3.2)	108 (32.5)
qS6	Has fallen in the last 6 months	Falls	171 (19.0)	49 (8.6)	122 (36.7)
qS7	Has difficulty swallowing or often coughs when drinking	Dysphagia	113 (12.6)	28 (4.3)	85 (25.6)
qS8	Lost a lot of weight in the past 6 months	Weight loss	176 (19.6)	58 (10.2)	118 (35.5)
qS9	Often feels down or depressed	Depression	257 (28.6)	110 (19.4)	147 (44.3)
qS10	Has incontinence problems and use pads to avoid getting wet	Incontinence	248 (27.6)	83 (14.6)	165 (49.7)
qS11	Suffers from insomnia	Insomnia	199 (22.1)	88 (15.5)	111 (33.4)
qS12	Takes sedatives or sleeping pills	Sedatives	254 (28.2)	116 (20.4)	138 (41.6)
qS13	Often complains of pain	Pain	264 (29.3)	135 (23.8)	129 (38.9)
qS14	Often feels weak and fatigued	Weakness	314 (34.9)	156 (27.5)	158 (47.6)
qS15	Has behavior problems	Behavior	55 (6.1)	11 (1.9)	44 (13.2)

All differences between groups are significant at *p* < 0.001.

### 2.3 Study design, participants and setting

This single-centre cross-sectional study involved a convenience sample of 900 community-dwelling patients aged over 50 years who were referred to our hospital for elective surgery or treatment of an acute medical condition. There were no other inclusion or exclusion criteria, except for patients who did not wish to participate in the study.

Of the 568 patients undergoing elective surgery (63.1% of the total sample), 286 (50.4%) were scheduled for urological procedures, 184 (32.4%) for hip or knee arthroplasty, and 98 (17.3%) for vascular surgery. The 332 patients with acute medical conditions (36.9% of the total sample) were admitted consecutively to the internal medicine unit from the emergency department of our hospital.

The IRCCS Humanitas Research Hospital is a tertiary teaching hospital accredited by the Joint Commission International. It uses integrated clinical pathways and an electronic medical record system to monitor healthcare professionals’ activities and ensure compliance with quality standards. These standards cover access to and continuity of care, patient assessment, care delivery, safety goals, anaesthesia and surgical care, medication management, and patient and family rights and education. The 40-bed internal medicine unit collaborates closely with the neurorehabilitation unit to share care pathways for frail and disabled patients.

### 2.4 Data collection

Patients scheduled for urological surgery completed the FLIGS-FQ online via a custom-built web platform. For all other patients, the FLIGS-FQ was collected during a face-to-face interview conducted by two trained interviewers. Elective surgical patients were interviewed at the time of their preoperative visit, while acute patients were interviewed within 48 h of being admitted to the internal medicine unit. Patients unable to participate fully in the interview were assisted by caregivers or family members. The average time taken to administer the FLIGS-FQ in person was 10 min. Data collection took place from 14 February 2022 to 31 October 2023.

### 2.5 Statistical analyses

Descriptive statistics were used to present demographic data and responses to the FLIGS-FQ for both elective and non-elective patients. Categorical variables are reported as counts and percentages, and the chi-square test or Fisher’s exact test was used to analyse differences between groups. Continuous variables are reported as the median and interquartile range (IQR), and the Wilcoxon rank sum test was used to analyse differences between groups. A two-tailed *p* < 0.05 was considered significant.

All statistical analyses, except CFA, were performed using Stata, version 18.5 (Stata Corp LLC, College Station, TX, USA). GraphPad Prism version 10.5.0 was used to create some graphs and perform a false discovery rate test in the differential item function analysis (see the IRT analysis section below).

#### 2.5.1 Psychometric validation

The psychometric validation of the FLIGS-FQ followed the COSMIN checklist (32). It was conducted in three steps.

First, CFA was used to verify the adequacy of the single-factor solution hypothesized for the initial pool of 23 FLIGS-FQ items. Second, both CFA and IRT were used in tandem to remove invalid items from the pool. At this step, items that violated local independence (i.e., showed a modification index greater than 10 in CFA) or invariance (i.e., differential item functioning in IRT) were considered candidates for removal. Items were removed by consensus, balancing the statistical fit of the model with the clinical soundness of the entire questionnaire. The third step involved using IRT exclusively to estimate the characteristics of each item, as well as the informative content and reliability of the entire FLIGS-FQ scale.

##### 2.5.1.1 CFA

CFA was conducted using lavaan (33), an R package included in JASP software (34). The WLSMV estimator with robust standard error calculation was used to account for categorical nature of the FLIGS-FQ items (35). Results are reported in accordance with current guidelines (36).

The following indices were used as references to assess the goodness-of-fit of the single-factor model (37):

1. *χ*^2^/df < 5;
2. root mean square error of approximation (RMSEA) ≤ 0.08;
3. standardised root mean square residual (SRMR) ≤ 0.08;
4. comparative fit index (CFI) > 0.90;
5. Tucker–Lewis index (TLI) ≥ 0.90.

Additionally, the internal consistency of the construct was assessed using McDonald’s Ω coefficient. Ω coefficient values between 0.70 and 0.79, as well as between 0.80 and 0.90, indicate acceptable and high internal consistency reliability, respectively.

##### 2.5.1.2 IRT analysis

The IRT analysis was carried out using the one-parameter logistic (1PL) model, also known as the Rasch model. Assuming identical discrimination for all item on a scale (i.e., the ability to distinguish individuals across all levels of the latent trait), the 1PL model estimates only the difficulty parameter of the items in order to determine an individual’s position on the latent theta trait scale. In the context of this study, the difficulty parameter can be interpreted as the degree of frailty required for an individual to respond positively to a question.

To obtain robust IRT estimates, bootstrap resampling with 1,000 replications was used, clustered according to patient group and gender. The differential item functioning was analysed by gender using the Mantel–Haenszel test. The false discovery rate was verified using the two-step procedure of Benjamini, Krieger and Yekutieli, set at 1% (38).

Results of the IRT analysis are reported quantitatively by presenting point estimates with 95% confidence intervals, and qualitatively by presenting diagnostic and analytical graphs of the item characteristic curves, test characteristic curve and test information function.

## 3 Results

### 3.1 Descriptive analysis

All completed questionnaires were included in the analysis, meaning that the FLIGS-FQ dataset contains no missing data.

The sample of 900 participants included 537 male patients (59.7%) and 363 female patients (40.3%). The median age of the entire sample was 73.4 years (IQR: 67.0–81.6) with an age range of 50–100 years. Acute medical patients were significantly older than elective surgical patients (median age: 81.5 years, IQR: 73.4–85. 8 years, vs. median age: 70.9 years, IQR: 66.0–76.0; *p* < 0.001) and had a significantly higher percentage of female patients (49.4% vs. 35.0%, *p* < 0.001).

Table 1 lists the 23 FLIGS-FQ questions and the frequency of their endorsement by elective and acute patients. Overall, endorsement of functional limitation items ranged from 14.2% (qF4, home mobility, and qF6, supervision) to 31.2% (qF7, accompaniment), whereas endorsement of syndromic items ranged from 6.1% (qS15, behavioral disturbance) to 46.1% (qS4, polypharmacy). Acute patients endorsed all 23 items to a much greater extent than elective patients, with all differences significant at *p* < 0.001.

The following sections report the main results of the psychometric validation. The full results can be found in the supplementary material.

### 3.2 Factorial validity and FLIGS-FQ refinement

The CFA demonstrated that the single-factor model fits the 23 items of the FLIGS-FQ sufficiently [*χ*^2^/df = 4.712; RMSEA = 0.064 (90% CI, 0.060–0.068); SRMR = 0.096; CFI = 0.967; TLI = 0.964]. However, a considerable amount of residual covariance and a coefficient of Ω = 0.926 indicated a level of item redundancy that could not be ignored.

Following the procedure described above, the following items were removed: qS4 (polypharmacy), qS11 (insomnia), qS12 (sedatives), qS14 (weakness), qF4 (home mobility), qF6 (supervision) and qF7 (accompaniment). It is important to note that items qS9 (depression) and qS13 (pain) were deliberately retained during the refinement of the FLIGS-FQ despite a moderate excess of covariance, as they were considered essential for a frailty screening tool.

Removing the invalid items resulted in a 16-item questionnaire, the FLIGS-FQ-16 (Supplementary Table 2), which fits the single-factor hypothesis much better than the initial pool of items while maintaining high internal consistency reliability [*χ*^2^/df = 3.001; RMSEA = 0.047 (90% CI, 0.041–0.053); SRMR = 0.075; CFI = 0.982; TLI = 0.979; Ω coefficient = 0.879].

**Figure 1:**
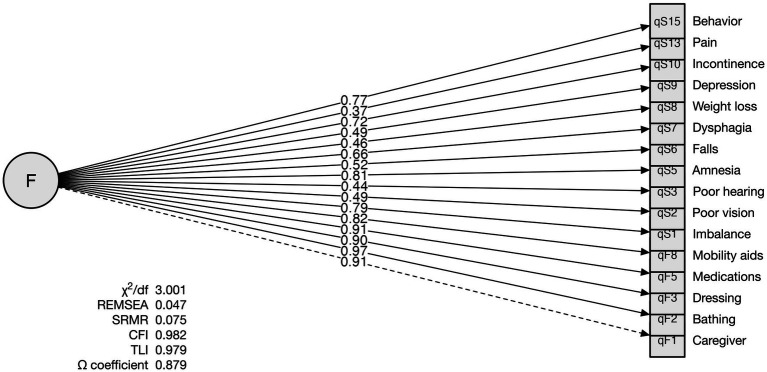
FLIGS-FQ-16 single-factor frailty model. The graph confirms the original hypothesis that frailty (F), conceptualized as a single continuous latent variable, is adequately represented by a set of items reflecting it (the rectangles). The directional arrow indicates the standardized frailty load for each item. The goodness-of-fit indices show the high reliability of the internal consistency of the construct.

For all items, the standardized factor loading was statistically significant and above the recommended threshold of 0.40, except for pain (0.37), indicating an adequate association between the item and frailty (Figure 1).

### 3.3 Item and test validity

**Figure 2:**
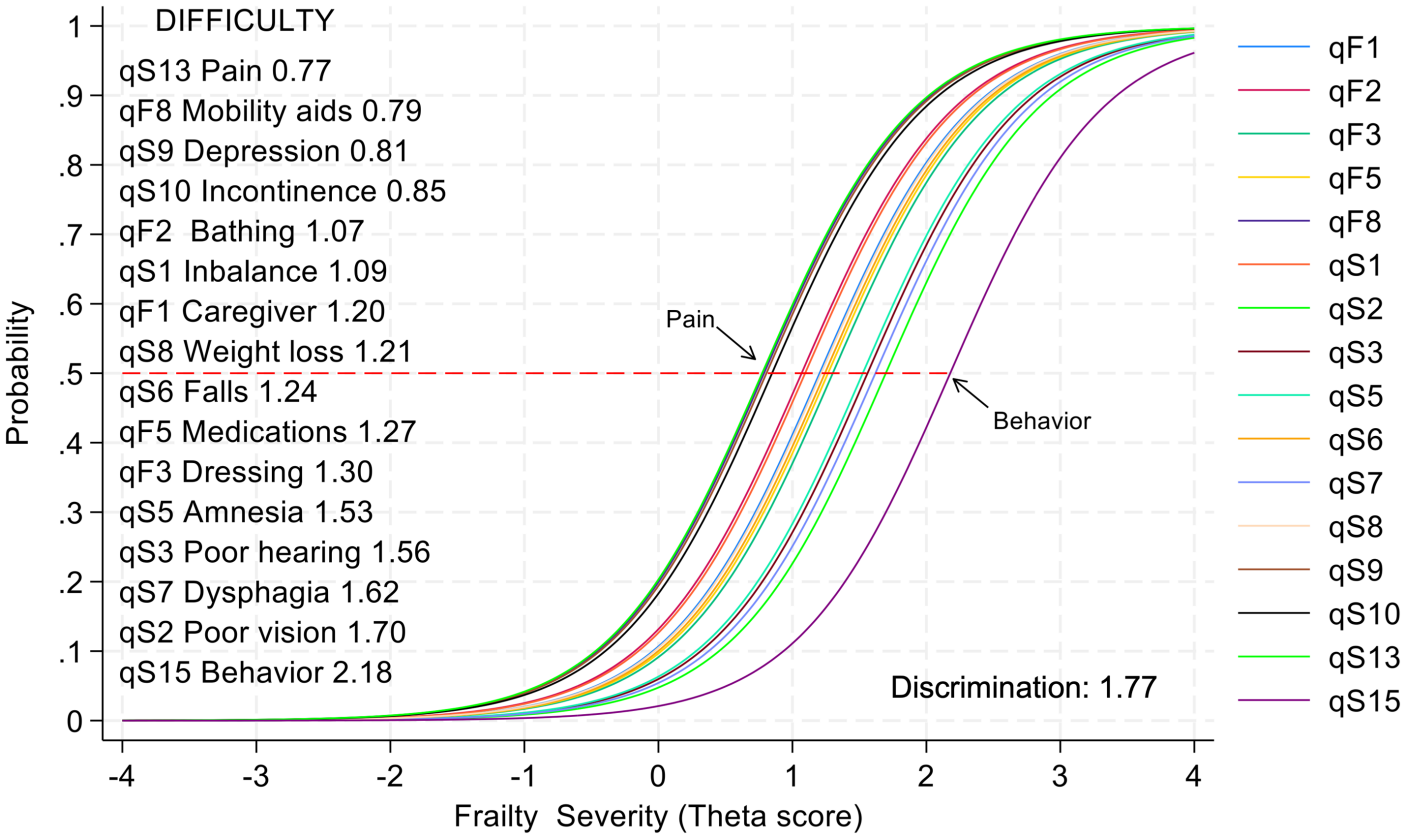
Item characteristic curves of the FLIGS-FQ-16. The curves illustrate the probability of each item occurring as a function of frailty severity (shifting to the right). The red dashed line indicates the 50% threshold and corresponds to the difficulty parameter of the listed items. Some curves overlap due to their very similar difficulties. The high common value of discrimination explains why the curves are so steep.

Item characteristic curves demonstrate that the probability of endorsing FLIGS-FQ-16 items increases monotonically as frailty severity increases. The good, shared discriminant value of 1.77 explains why the curves are so steep. The difficulty of the items covers a range of frailty severity from 0.77 (pain) to 2.18 (behavioural disorders) (Figure 2).

**Figure 3:**
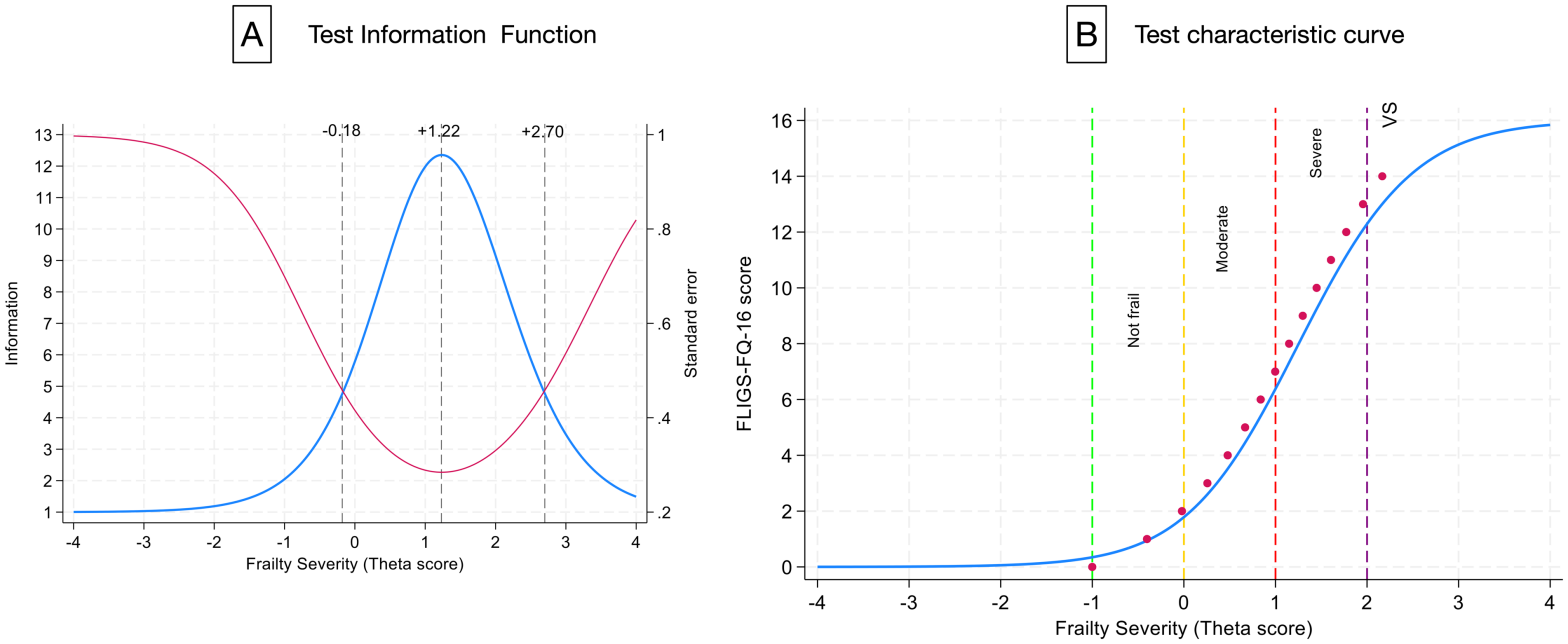
FLIGS-FQ-16 test reliability. **(A)** Shows the accuracy of the information from the entire test (blue line), which is obtained by adding the probabilities of the individual items together. The vertical dashed lines indicate the boundaries of the information. The greater the distance from the information peak in either direction, the greater the standard error (red line) and the less information the instrument provides about a person’s frailty. **(B)** Illustrates the strong calibration between the predicted frailty theta score (blue line) and the FLIGS-FQ-16 scores (red dots). The vertical dashed lines indicate the FLIGS-FQ-16 score threshold for standardized patient classification. The cut-off value of 3 separates progressively more frail patients from non-frail patients. VS, very severe.

The entire test provides reliable information on frailty severity for values between −0.18 to +2.70 standard deviations from the population mean, peaking at +1.22 (Figure 3A). The good calibration between FLIGS-FQ-16 scores and the theta frailty score estimated by IRT using the Bayesian empirical estimator reliably classifies patients along the frailty severity continuum (Figure 3B).

### 3.4 Patient classification

The total FLIGS-FQ-16 score in the entire sample ranged from 0 to 14 according to increasing severity of frailty, with a median value of 2 (IQR: 0–5). Based on the IRT test characteristic curve, patients with FLIGS-FQ-16 scores from 0 to 2 were classified as “non-frail” (i.e., within one standard deviation below the population mean), and those with scores from 3 to 7 were classified as “moderately frail” (i.e., within one standard deviation above the population mean). Those with scores ranging from 8 to 13, as well as those with a score of 14, were classified as “severely frail” or “very severely frail” (i.e., within or beyond two standard deviations above the population mean). The only two patients with a score of 14 were included in the “severely frail” category.

**Figure 4:**
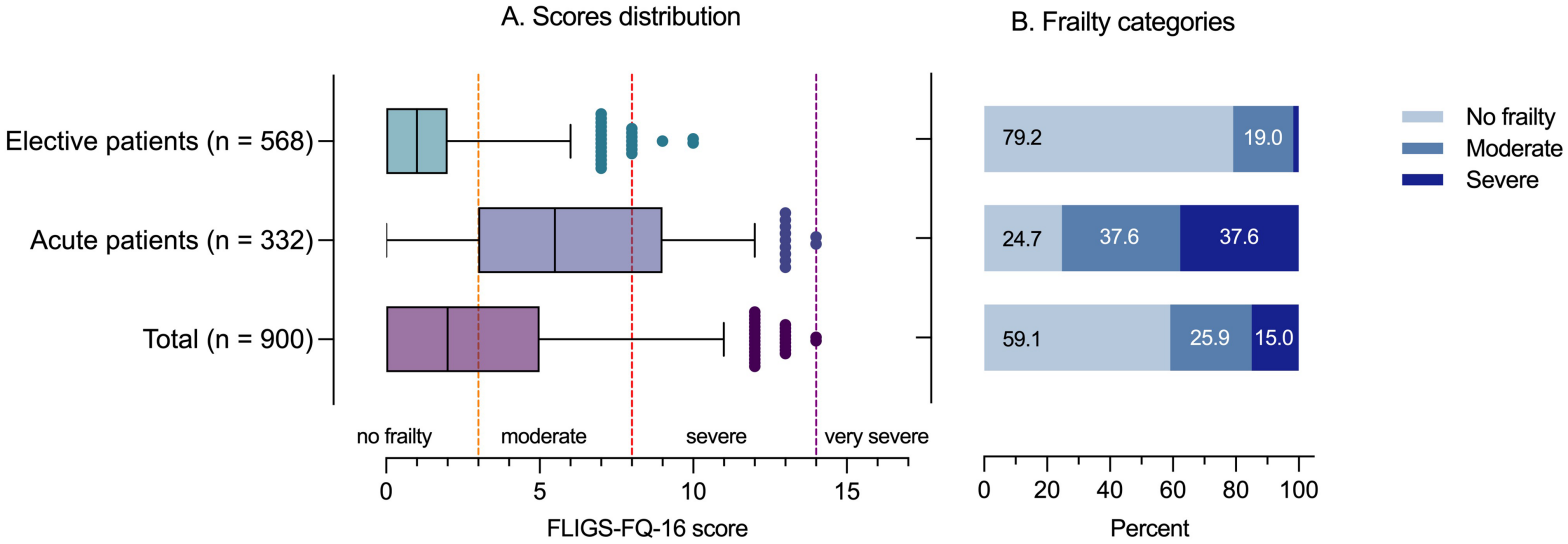
FLIGS-FQ-16 score and frailty categories among elective surgical and acute medical patients. **(A)** Shows the median value and interquartile range in the boxes, and the whiskers show the 5th and 95th percentile values. The dots represent outliers. The vertical dashed lines indicate the threshold values for uniformly classifying frailty. **(B)** Shows the resulting frailty categories.

Overall, 532 patients (59.1%) were classified as “non-frail,” 233 patients (25.9%) as “moderately frail,” and 135 patients (15.0%) as “severely frail.” Figure 4 shows the distribution of the total FLIGS-FQ-16 score and the resulting frailty classification of elective and acute patients. Acute patients had significantly higher FLIGS-FQ-16 scores than elective patients (median: 5.5; IQR: 3–9 vs. median: 1; IQR: 0–2; *p* < 0.001), resulting in a significantly higher prevalence of frail patients (75.3% vs. 20.8%, *p* < 0.001). Among acute patients, 37.6% were moderately or severely frail. Among elective patients, 19.0% were moderately frail and 1.8% were severely frail.

## 4 Discussion

This paper presents the development and psychometric validation results of the FLIGS-FQ-16, a new, multidomain questionnaire designed to assess frailty in hospital settings.

The FLIGS-FQ-16 includes 16 indicators, five of which are functional limitations related to instrumental (caregiver and medication management) and basic (bathing, dressing, and using mobility aids) activities of daily living. The remaining 11 indicators are geriatric syndromes related to mental and sensorial health (amnesia, behavioral disorders, poor vision, and poor hearing), psychological health (depression), and physical health (imbalance, falls, dysphagia, weight loss, urinary incontinence, and pain).

To the best of our knowledge, the FLIGS-FQ-16 is the first frailty measurement tool to have undergone comprehensive psychometric validation according to modern standards.

### 4.1 Rationale

Consistent with existing literature on the subject, this study offers a novel interpretation of frailty as a “syndrome of syndromes.” The FLIGS-FQ-16 framework conceptualized frailty is a single, continuous latent trait that can be quantified using reflective indicators, such as functional limitations and geriatric syndromes. Psychometric validation of this model produced an interval scale capable of measuring frailty up to its most severe levels.

Functional limitations and geriatric syndromes are widely recognized as higher-order manifestations of frailty that capture the combined effects of aging and diseases (39). These items have received substantial endorsement for use in screening for frailty in emergency departments (40) and are incorporated into all valid instruments that refer to a multidimensional construct of frailty, albeit in disparate ways (13, 15, 41, 42).

We deliberately excluded specific diseases and social frailty from the FLIGS-FQ-16 construct under the assumption that they are causal factors of frailty with highly variable effects on patient diagnosis and prognosis. Multimorbidity and social frailty are undoubtedly fundamental components of a comprehensive geriatric assessment. However, we believe that these dimensions should be assessed independently and, in the case of multimorbidity, with much greater analytical rigor than is currently permitted by a heterogeneous list of conditions.

### 4.2 Psychometric validity

The reliability of the FLIGS-FQ-16 construct, and derived metrics has been extensively demonstrated by psychometric validation. This allows for full mastery of the instrument for diagnostic and prognostic purposes.

Confirmatory factor analysis showed that the single-factor model of the FLIGS-FQ-16 had satisfactory fit indices (*χ*^2^/df = 3.001, RMSEA = 0.047, SRMR = 0.075, CFI = 0.982, TLI = 0.979). Furthermore, McDonald’s Ω coefficient= 0.879 revealed a high degree of internal consistency, confirming that all scale items are positively correlated and consistently measure the same underlying construct. All FLIGS-FQ-16 items except pain (0.37) had frailty load values above the recommended threshold of 0.40 indicating an adequate association between frailty and each item.

Item response theory demonstrated the reliability of our instrument at the item and scale levels. The FLIGS-FQ-16 consists of items with an equal function for both sexes and high common discrimination of 1.77. The items vary in difficulty from 0.77 (pain) to 2.18 (behavioral disturbances) standard deviations above the population mean.

The total FLIGS-FQ-16 score reliably provides information on the severity of individual frailty, ranging from 0.18 standard deviations below the population mean (not frail) to 2.7 standard deviations above the population mean (very severely frail). This allows patients to be consistently classified. FLIGS-FQ-16 scores 0–2 identify non-frail patients, and thresholds of 3, 8 and 14 distinguish between those who are moderately, severely and very severely frail, respectively.

### 4.3 Translating FLIGS-FQ information into routine care

The FLIGS-FQ model quantifies individual frailty by flagging potentially reversible risk factors that constitute specific treatment targets. All 23 items of the FLIGS FQ can be used to obtain a comprehensive collection of patient history data, bearing in mind that seven items (polypharmacy, insomnia, sedatives, weakness, home mobility, supervision, accompaniment) are not psychometrically valid, as they duplicate information provide by other items. Nevertheless, items such as polypharmacy and the use of sleeping pills or tranquillisers may still constitute a clinically significant alert.

Our findings confirm the effectiveness of FLIGS-FQ-16 as a tool for frailty screening and decision-making in perioperative medicine and the management of older adult in hospital. These are two important areas of geriatric co-management where evidence is limited and treatments are still poorly defined (43, 44).

Οf patient undergoing elective surgery, 20.8% had geriatric syndromes and functional limitations classifying them as moderately (19.0%) or severely (1.8%) frail. For these patients, the FLIGS-FQ-16 could inform decisions aimed at mitigating the impact of surgery and establishing proactive perioperative care pathways tailored to individual frailty (45). This would represent an improvement on the current provision of undifferentiated physical exercise and nutritional programmes, particularly for the frailest patients.

In contrast, 75.3% of non-elective patients admitted for medical illness were classified as frail, which is consistent with the higher prevalence reported in previous studies (46). Among our patients, 37.6% were moderately frail, and 37.6% were severely frail. This high prevalence and severity of frailty is undoubtedly due to the high premorbid burden of geriatric syndromes and disabilities, as well as advanced age.

This clinical scenario strongly questions the feasibility and adequacy of the “universal” approach recommended for frail hospitalized patients. This approach remains exclusively based on physical exercise, nutritional optimization, and other generic recommendations (47–49). Older patients admitted to hospital for acute medical conditions need intensive and targeted medical and functional workups, as well as appropriated multimodal rehabilitation treatments. Best practice guidelines clearly indicate that rehabilitation treatments effectively alleviate functional limitations and geriatric syndromes with varying degrees of focus and intensity.

### 4.4 Standardizing care and outcomes

The FLIGS-FQ-16 framework can integrate best care practices into personalized care plans, even for the frailest patients. In addition to initiating specific diagnostic and therapeutic processes for each positive screening result, qualitative interpretation of each patient’s FLIGS-FQ-16 profile enables the establishment of personalized treatment goals and timelines, as well as the definition of geriatric team professionals’ roles in their management. Standardizing assessment and treatment processes enables workflow monitoring, result verification, and intervention adequacy and effectiveness comparison at all levels of frailty severity.

### 4.5 Transferability and generalizability

The FLIGS-FQ-16 is a patient-reported outcome measure with good face and psychometric validity. Its items adequately cover the range of clinical problems encountered in geriatric practice. These characteristics make it suitable for use in various hospital settings. For example, it is particularly useful in oncology, providing meaningful information to guide decisions regarding chemotherapy or invasive procedures, and triggering specific interventions to minimize iatrogenic impact.

However, further validation is required when transferring it across different care settings. This should include psychometric validation to verify construct stability and item accuracy, followed by prognostic validation to consider adverse health outcomes associated with frailty thresholds.

The same validation process should be repeated to ensure broader generalizability of the FLIGS-FQ-16. The convenience sampling strategy used may have introduced bias, particularly given the overrepresentation of urological patients in the elective group. To obtain definitive estimates, future studies should use stratified or population-based sampling.

### 4.6 Prognostic value

The FLIGS-FQ-16 score meets all the basic criteria required for it to be used as a high-quality prognostic research tool. You can use it either independently or in tandem with calibrated sets of causal items of frailty, such as in fact multi-morbidities and social and environmental determinants. It can also be used with condition-specific indicators; for example, with cancer patients.

Testing and tracking each dimension of frailty separately helps us understand how they interact with each other and how their prognostic value changes over time. This provides a more detailed picture of how different predictors impact various outcomes at different points along the frailty trajectory.

We are currently analysing the prognostic value of the FLIGS-FQ-16 score in predicting adverse health outcomes in the group of acute patients participating in this study. We are examining how it performs as both a continuous and a discretized variable in relation to identified frailty thresholds. Although these analyses do not include direct comparisons with other frailty assessment tools, it is still possible to speculate about how well the FLIGS-FQ-16 performs in predicting adverse hospital outcomes compared to other tools.

We expect the FLIGS-FQ-16 to perform equally or slightly worse than instruments that incorporate multimorbidity items, either directly or indirectly, such as the Frailty Index (50) or the Clinical Frailty Scale (51) with regard to disease-related outcomes. These include in-hospital morbidity and mortality, as well as unplanned readmissions. However, we anticipate that the FLIGS-FQ-16 will significantly outperform existing instruments in terms of patient-related outcomes, such as prolonged hospital stays, disability at discharge, and non-home discharges.

### 4.7 Strengths and limitations

This study has two key strengths. Firstly, data from two groups of patients with very different characteristics enabled us to validate our tool across the entire expected score range. Secondly, the large sample size ensured the results were statistically valid and reliable.

The limitations are as follows. Firstly, since the FLIGS-FQ project involved psychometric and clinimetric validation of the instrument without comparison to other frailty instruments, the FLGS-FQ-16 lacks concurrent validity. This should be prioritized for future work. Secondly, the cross-sectional design required for psychometric validation prevents an understanding of frailty trajectories, which are essential for mitigating the condition in a clinical setting. Thirdly, unlike many other scales that claim to measure it, the FLIGS-FQ-16 does not provide information on the so-called “pre-frailty” category.

## 5 Conclusion

The FLIGS-FQ-16 is a multidomain frailty questionnaire based on treatable risk factors. Its psychometric validation from a single-factor perspective has produced a common frailty metric, enabling consistent patient classification according to their level of frailty. These characteristics make the FLIGS-FQ-16 an effective screening tool for identifying vulnerable individuals in hospital settings. The FLIGS-FQ-16 is also an important resource for developing personalized treatment plans and standardizing decision-making processes in complex multidisciplinary contexts.

We hope that FLIGS-FQ-16 will encourage the development of banks containing psychometrically valid items to create new-generation metrics that will improve the accuracy of preventive and rehabilitative interventions for frailty.

## Data Availability

The raw data supporting the conclusions of this article will be made available by the authors, without undue reservation.
