# Supplementary material for: Scaling up frailty: psychometric validation of the functional limitations and geriatric syndromes frailty questionnaire—a new tool for uniformly classifying vulnerable hospital patients
Source: Front Med (Lausanne). 2025 Oct 3;12:1642562. doi: 10.3389/fmed.2025.1642562 (PMC12531211; doi:10.3389/fmed.2025.1642562)
Supplement: Supplementary file 1 [file Table_1.DOCX]

PSYCHOMETRIC VALIDATION OF THE FUNCTIONAL LIMITATIONS AND GERIATRIC SYNDROMES FRAILTY QUESTIONNAIRE

| **Table 1. Multi-domain arrangement of the 23 items of FLIGS-FQ** | | | |
| --- | --- | --- | --- |
|  |  |  |  |
|  |  | **Short label** |  |
|  |  |  |  |
|  |  |  |  |
| **Limitation in I-ADL** |  | qF1. Caregiver |  |
|  |  | qF5. Medications |  |
|  |  | qF6. Supervision |  |
| **Limitations in B-ADL** |  | qF2. Bathing |  |
|  |  | qF3. Dressing |  |
| Mobility |  | qF4. Home mobility |  |
|  |  | qF7. Accompaniment |  |
|  |  | qF8. Mobility aids |  |
| **Mental and Sensorial** |  | qS5. Amnesia |  |
|  |  | qS15. Behavior |  |
|  |  | qS2. Poor vision |  |
|  |  | qS3. Poor hearing |  |
| **Psychological** |  | qS9. Depression |  |
|  |  |  |  |
| **Physical function** |  |  |  |
| Balance and falls |  | qS1. Imbalance |  |
|  |  | qS6. Falls |  |
| Weakness |  | qS14. Weakness |  |
| Weight |  | qS8. Weight loss |  |
| Incontinence |  | qS10. Incontinence |  |
| Dysphagia |  | qS7. Dysphagia |  |
| Insomnia |  | qS11. Insomnia |  |
| Pain |  | qS13. Pain |  |
|  |  |  |  |
| **Multimorbidity** |  | qS4. Polypharmacy |  |
|  |  | qS12. Sedatives |  |
|  |  |  |  |

| **Tetrachoric correlation coefficients between the 23 items of FLIGS-FQ** |
| --- |
| 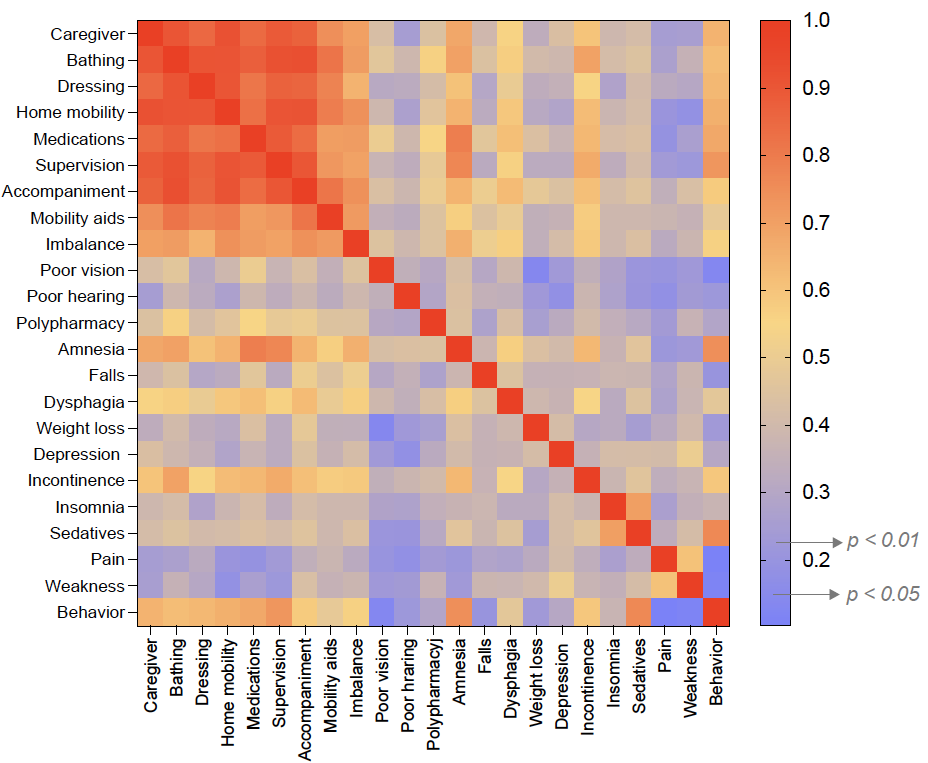 |
|  |
| As shown in the heat map, the items are positively associated with each other at highly significant levels, with only a few exceptions. This positive interrelationship is a fundamental characteristic of single-factor reflective models. |

**Confirmatory Factor Analysis (CFA) of FLIGS-FQ-23 items**

Estimator: WLSMV; Standard error calculation: robust.

**Model ploτ**


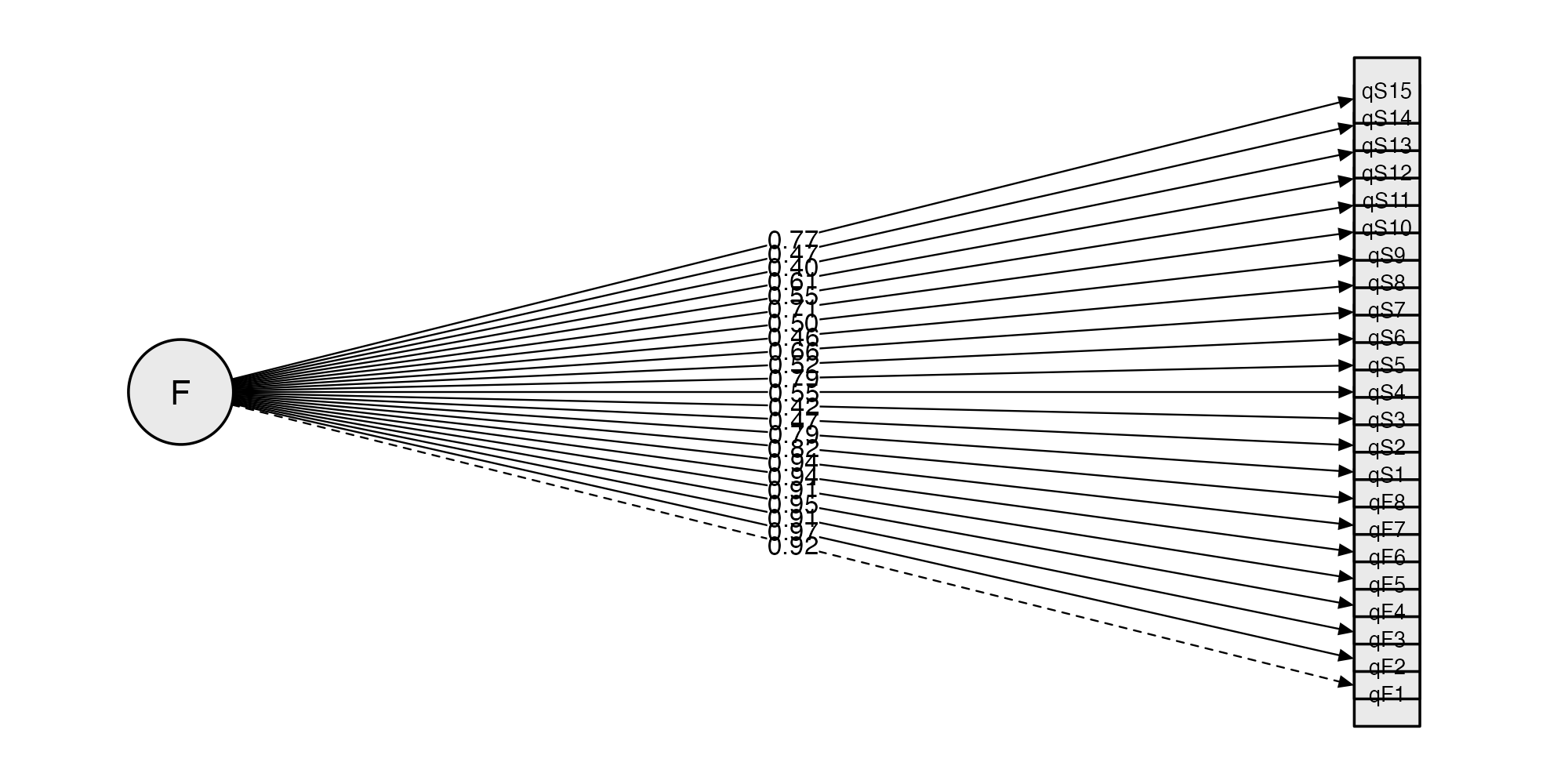


The plot illustrates the relationships between the single hidden variable (frailty, F) and the 23 items of the FLIGS-FQ (the rectangles). The factor loadings are standardized.

**Model Fit**

| *Chi-square test* | | | | | | | |
| --- | --- | --- | --- | --- | --- | --- | --- |
| Model | | Χ² | | df | | p | |
| Baseline model |  | 26174.327 |  | 253 |  |  |  |
| Factor model |  | 1083.755 |  | 230 |  | < .001 |  |
|  | | | | | | | |

| *Fit indices* | | | |
| --- | --- | --- | --- |
| Index | | Value | |
| Comparative Fit Index (CFI) |  | 0.967 |  |
| Tucker-Lewis Index (TLI) |  | 0.964 |  |
| Bentler-Bonett Non-normed Fit Index (NNFI) |  | 0.964 |  |
| Bentler-Bonett Normed Fit Index (NFI) |  | 0.959 |  |
| Parsimony Normed Fit Index (PNFI) |  | 0.895 |  |
| Bollen’s Relative Fit Index (RFI) |  | 0.954 |  |
| Bollen’s Incremental Fit Index (IFI) |  | 0.967 |  |
| Relative Noncentrality Index (RNI) |  | 0.967 |  |
|  | | | |
| *Note.*  Except for the PNFI, the fit indices are scaled because of categorical variables in the data. | | | |

| *Other fit measures* | | | |
| --- | --- | --- | --- |
| Metric | | Value | |
| Root mean square error of approximation (RMSEA) |  | 0.064 |  |
| RMSEA 90% CI lower bound |  | 0.060 |  |
| RMSEA 90% CI upper bound |  | 0.068 |  |
| RMSEA p-value |  | 7.908×10^-10^ |  |
| Standardized root mean square residual (SRMR) |  | 0.096 |  |
| Hoelter’s critical N (α = .05) |  | 266.360 |  |
| Hoelter’s critical N (α = .01) |  | 282.733 |  |
| Goodness of fit index (GFI) |  | 0.986 |  |
| McDonald fit index (MFI) |  | 0.688 |  |
| Expected cross validation index (ECVI) |  |  |  |
|  | | | |
| *Note.*  The RMSEA results are scaled because of categorical variables in the data. | | | |

| *Average variance extracted* | | | |
| --- | --- | --- | --- |
| Factor | | AVE | |
| F |  | 0.524 |  |
|  | | | |

| *Reliability* | | | | | |
| --- | --- | --- | --- | --- | --- |
|  | | Coefficient ω | | Coefficient α | |
| F |  | 0.926 |  | 0.896 |  |
|  | | | | | |

**Parameter estimates**

| *Factor loadings* | | | | | | | | | | | | | | | |
| --- | --- | --- | --- | --- | --- | --- | --- | --- | --- | --- | --- | --- | --- | --- | --- |
|  | | | | | | | | | | | | 95% Confidence Interval | | | |
| Factor | | Indicator | | Estimate | | Std. Error | | z-value | | p | | Lower | | Upper | |
| F |  | qF1 |  | 1.000 |  | 0.000 |  |  |  |  |  | 1.000 |  | 1.000 |  |
|  |  | qF2 |  | 1.047 |  | 0.016 |  | 66.987 |  | < .001 |  | 1.017 |  | 1.078 |  |
|  |  | qF3 |  | 0.984 |  | 0.019 |  | 51.841 |  | < .001 |  | 0.947 |  | 1.021 |  |
|  |  | qF4 |  | 1.029 |  | 0.018 |  | 58.033 |  | < .001 |  | 0.994 |  | 1.063 |  |
|  |  | qF5 |  | 0.983 |  | 0.019 |  | 51.795 |  | < .001 |  | 0.946 |  | 1.021 |  |
|  |  | qF6 |  | 1.021 |  | 0.017 |  | 61.595 |  | < .001 |  | 0.989 |  | 1.054 |  |
|  |  | qF7 |  | 1.023 |  | 0.017 |  | 60.355 |  | < .001 |  | 0.990 |  | 1.057 |  |
|  |  | qF8 |  | 0.890 |  | 0.026 |  | 34.835 |  | < .001 |  | 0.840 |  | 0.940 |  |
|  |  | qS1 |  | 0.851 |  | 0.028 |  | 30.345 |  | < .001 |  | 0.796 |  | 0.906 |  |
|  |  | qS2 |  | 0.511 |  | 0.053 |  | 9.569 |  | < .001 |  | 0.406 |  | 0.615 |  |
|  |  | qS3 |  | 0.458 |  | 0.053 |  | 8.695 |  | < .001 |  | 0.355 |  | 0.562 |  |
|  |  | qS4 |  | 0.598 |  | 0.041 |  | 14.549 |  | < .001 |  | 0.517 |  | 0.678 |  |
|  |  | qS5 |  | 0.853 |  | 0.031 |  | 27.513 |  | < .001 |  | 0.792 |  | 0.913 |  |
|  |  | qS6 |  | 0.559 |  | 0.046 |  | 12.183 |  | < .001 |  | 0.469 |  | 0.649 |  |
|  |  | qS7 |  | 0.719 |  | 0.043 |  | 16.812 |  | < .001 |  | 0.635 |  | 0.803 |  |
|  |  | qS8 |  | 0.502 |  | 0.048 |  | 10.476 |  | < .001 |  | 0.408 |  | 0.596 |  |
|  |  | qS9 |  | 0.543 |  | 0.044 |  | 12.216 |  | < .001 |  | 0.456 |  | 0.631 |  |
|  |  | qS10 |  | 0.767 |  | 0.033 |  | 23.058 |  | < .001 |  | 0.701 |  | 0.832 |  |
|  |  | qS11 |  | 0.600 |  | 0.044 |  | 13.644 |  | < .001 |  | 0.514 |  | 0.686 |  |
|  |  | qS12 |  | 0.664 |  | 0.038 |  | 17.309 |  | < .001 |  | 0.589 |  | 0.740 |  |
|  |  | qS13 |  | 0.438 |  | 0.047 |  | 9.260 |  | < .001 |  | 0.345 |  | 0.530 |  |
|  |  | qS14 |  | 0.505 |  | 0.046 |  | 10.980 |  | < .001 |  | 0.415 |  | 0.595 |  |
|  |  | qS15 |  | 0.830 |  | 0.049 |  | 17.062 |  | < .001 |  | 0.735 |  | 0.925 |  |
|  | | | | | | | | | | | | | | | |

| *Factor loadings* | | | | | | | | | | | | | | | |
| --- | --- | --- | --- | --- | --- | --- | --- | --- | --- | --- | --- | --- | --- | --- | --- |
|  | | | | | | | | | | | | 95% Confidence Interval | | | |
| Factor | | Indicator | | Std. Estimate | | Std. Error | | z-value | | p | | Lower | | Upper | |
| F |  | qF1 |  | 0.923 |  | 0.013 |  | 71.437 |  | < .001 |  | 0.897 |  | 0.948 |  |
|  |  | qF2 |  | 0.966 |  | 0.008 |  | 121.333 |  | < .001 |  | 0.951 |  | 0.982 |  |
|  |  | qF3 |  | 0.908 |  | 0.015 |  | 60.435 |  | < .001 |  | 0.879 |  | 0.937 |  |
|  |  | qF4 |  | 0.949 |  | 0.013 |  | 73.994 |  | < .001 |  | 0.924 |  | 0.974 |  |
|  |  | qF5 |  | 0.907 |  | 0.015 |  | 59.677 |  | < .001 |  | 0.878 |  | 0.937 |  |
|  |  | qF6 |  | 0.943 |  | 0.012 |  | 79.523 |  | < .001 |  | 0.919 |  | 0.966 |  |
|  |  | qF7 |  | 0.944 |  | 0.010 |  | 90.270 |  | < .001 |  | 0.924 |  | 0.965 |  |
|  |  | qF8 |  | 0.821 |  | 0.022 |  | 37.557 |  | < .001 |  | 0.778 |  | 0.864 |  |
|  |  | qS1 |  | 0.785 |  | 0.026 |  | 30.515 |  | < .001 |  | 0.735 |  | 0.836 |  |
|  |  | qS2 |  | 0.471 |  | 0.049 |  | 9.635 |  | < .001 |  | 0.375 |  | 0.567 |  |
|  |  | qS3 |  | 0.423 |  | 0.049 |  | 8.710 |  | < .001 |  | 0.328 |  | 0.518 |  |
|  |  | qS4 |  | 0.551 |  | 0.038 |  | 14.477 |  | < .001 |  | 0.477 |  | 0.626 |  |
|  |  | qS5 |  | 0.787 |  | 0.028 |  | 27.702 |  | < .001 |  | 0.731 |  | 0.842 |  |
|  |  | qS6 |  | 0.516 |  | 0.041 |  | 12.530 |  | < .001 |  | 0.435 |  | 0.596 |  |
|  |  | qS7 |  | 0.663 |  | 0.039 |  | 17.026 |  | < .001 |  | 0.587 |  | 0.740 |  |
|  |  | qS8 |  | 0.463 |  | 0.044 |  | 10.573 |  | < .001 |  | 0.377 |  | 0.549 |  |
|  |  | qS9 |  | 0.502 |  | 0.041 |  | 12.351 |  | < .001 |  | 0.422 |  | 0.581 |  |
|  |  | qS10 |  | 0.707 |  | 0.030 |  | 23.426 |  | < .001 |  | 0.648 |  | 0.767 |  |
|  |  | qS11 |  | 0.554 |  | 0.040 |  | 13.753 |  | < .001 |  | 0.475 |  | 0.633 |  |
|  |  | qS12 |  | 0.613 |  | 0.035 |  | 17.425 |  | < .001 |  | 0.544 |  | 0.682 |  |
|  |  | qS13 |  | 0.404 |  | 0.043 |  | 9.347 |  | < .001 |  | 0.319 |  | 0.488 |  |
|  |  | qS14 |  | 0.466 |  | 0.041 |  | 11.311 |  | < .001 |  | 0.385 |  | 0.547 |  |
|  |  | qS15 |  | 0.766 |  | 0.045 |  | 16.969 |  | < .001 |  | 0.678 |  | 0.854 |  |
|  | | | | | | | | | | | | | | | |

| *Residual variances* | | | | | | | | | | | | | |
| --- | --- | --- | --- | --- | --- | --- | --- | --- | --- | --- | --- | --- | --- |
|  | | | | | | | | | | 95% Confidence Interval | | | |
| Indicator | | Estimate | | Std. Error | | z-value | | p | | Lower | | Upper | |
| qF1 |  | 0.148 |  | 0.000 |  |  |  |  |  | 0.148 |  | 0.148 |  |
| qF2 |  | 0.066 |  | 0.000 |  |  |  |  |  | 0.066 |  | 0.066 |  |
| qF3 |  | 0.176 |  | 0.000 |  |  |  |  |  | 0.176 |  | 0.176 |  |
| qF4 |  | 0.099 |  | 0.000 |  |  |  |  |  | 0.099 |  | 0.099 |  |
| qF5 |  | 0.177 |  | 0.000 |  |  |  |  |  | 0.177 |  | 0.177 |  |
| qF6 |  | 0.111 |  | 0.000 |  |  |  |  |  | 0.111 |  | 0.111 |  |
| qF7 |  | 0.108 |  | 0.000 |  |  |  |  |  | 0.108 |  | 0.108 |  |
| qF8 |  | 0.326 |  | 0.000 |  |  |  |  |  | 0.326 |  | 0.326 |  |
| qS1 |  | 0.383 |  | 0.000 |  |  |  |  |  | 0.383 |  | 0.383 |  |
| qS2 |  | 0.778 |  | 0.000 |  |  |  |  |  | 0.778 |  | 0.778 |  |
| qS3 |  | 0.821 |  | 0.000 |  |  |  |  |  | 0.821 |  | 0.821 |  |
| qS4 |  | 0.696 |  | 0.000 |  |  |  |  |  | 0.696 |  | 0.696 |  |
| qS5 |  | 0.381 |  | 0.000 |  |  |  |  |  | 0.381 |  | 0.381 |  |
| qS6 |  | 0.734 |  | 0.000 |  |  |  |  |  | 0.734 |  | 0.734 |  |
| qS7 |  | 0.560 |  | 0.000 |  |  |  |  |  | 0.560 |  | 0.560 |  |
| qS8 |  | 0.785 |  | 0.000 |  |  |  |  |  | 0.785 |  | 0.785 |  |
| qS9 |  | 0.748 |  | 0.000 |  |  |  |  |  | 0.748 |  | 0.748 |  |
| qS10 |  | 0.500 |  | 0.000 |  |  |  |  |  | 0.500 |  | 0.500 |  |
| qS11 |  | 0.693 |  | 0.000 |  |  |  |  |  | 0.693 |  | 0.693 |  |
| qS12 |  | 0.624 |  | 0.000 |  |  |  |  |  | 0.624 |  | 0.624 |  |
| qS13 |  | 0.837 |  | 0.000 |  |  |  |  |  | 0.837 |  | 0.837 |  |
| qS14 |  | 0.783 |  | 0.000 |  |  |  |  |  | 0.783 |  | 0.783 |  |
| qS15 |  | 0.413 |  | 0.000 |  |  |  |  |  | 0.413 |  | 0.413 |  |
|  | | | | | | | | | | | | | |

| *Thresholds* | | | | | | | | | | | | | | | |
| --- | --- | --- | --- | --- | --- | --- | --- | --- | --- | --- | --- | --- | --- | --- | --- |
|  | | | | | | | | | | | | 95% Confidence Interval | | | |
| Indicator | | Threshold | | Estimate | | Std. Error | | z-value | | p | | Lower | | Upper | |
| qF1 |  | t1 |  | 0.850 |  | 0.048 |  | 17.784 |  | 0.000 |  | 0.756 |  | 0.943 |  |
| qF2 |  | t1 |  | 0.754 |  | 0.046 |  | 16.236 |  | 0.000 |  | 0.663 |  | 0.845 |  |
| qF3 |  | t1 |  | 0.920 |  | 0.049 |  | 18.805 |  | 0.000 |  | 0.824 |  | 1.015 |  |
| qF4 |  | t1 |  | 1.070 |  | 0.052 |  | 20.671 |  | 0.000 |  | 0.969 |  | 1.172 |  |
| qF5 |  | t1 |  | 0.894 |  | 0.048 |  | 18.448 |  | 0.000 |  | 0.799 |  | 0.989 |  |
| qF6 |  | t1 |  | 1.070 |  | 0.052 |  | 20.671 |  | 0.000 |  | 0.969 |  | 1.172 |  |
| qF7 |  | t1 |  | 0.490 |  | 0.044 |  | 11.210 |  | 0.000 |  | 0.404 |  | 0.575 |  |
| qF8 |  | t1 |  | 0.553 |  | 0.044 |  | 12.517 |  | 0.000 |  | 0.467 |  | 0.640 |  |
| qS1 |  | t1 |  | 0.772 |  | 0.047 |  | 16.549 |  | 0.000 |  | 0.681 |  | 0.864 |  |
| qS2 |  | t1 |  | 1.203 |  | 0.055 |  | 21.921 |  | 0.000 |  | 1.096 |  | 1.311 |  |
| qS3 |  | t1 |  | 1.106 |  | 0.053 |  | 21.040 |  | 0.000 |  | 1.003 |  | 1.209 |  |
| qS4 |  | t1 |  | 0.098 |  | 0.042 |  | 2.332 |  | 0.020 |  | 0.016 |  | 0.180 |  |
| qS5 |  | t1 |  | 1.080 |  | 0.052 |  | 20.778 |  | 0.000 |  | 0.978 |  | 1.182 |  |
| qS6 |  | t1 |  | 0.878 |  | 0.048 |  | 18.208 |  | 0.000 |  | 0.783 |  | 0.972 |  |
| qS7 |  | t1 |  | 1.148 |  | 0.054 |  | 21.444 |  | 0.000 |  | 1.043 |  | 1.253 |  |
| qS8 |  | t1 |  | 0.858 |  | 0.048 |  | 17.905 |  | 0.000 |  | 0.764 |  | 0.951 |  |
| qS9 |  | t1 |  | 0.566 |  | 0.044 |  | 12.777 |  | 0.000 |  | 0.480 |  | 0.653 |  |
| qS10 |  | t1 |  | 0.596 |  | 0.045 |  | 13.361 |  | 0.000 |  | 0.509 |  | 0.684 |  |
| qS11 |  | t1 |  | 0.768 |  | 0.047 |  | 16.487 |  | 0.000 |  | 0.677 |  | 0.860 |  |
| qS12 |  | t1 |  | 0.576 |  | 0.044 |  | 12.972 |  | 0.000 |  | 0.489 |  | 0.663 |  |
| qS13 |  | t1 |  | 0.544 |  | 0.044 |  | 12.321 |  | 0.000 |  | 0.457 |  | 0.630 |  |
| qS14 |  | t1 |  | 0.388 |  | 0.043 |  | 9.038 |  | 0.000 |  | 0.304 |  | 0.473 |  |
| qS15 |  | t1 |  | 1.546 |  | 0.066 |  | 23.378 |  | 0.000 |  | 1.416 |  | 1.675 |  |
|  | | | | | | | | | | | | | | | |

### Modification Indices

| *Residual covariances* | | | | | | | | | |
| --- | --- | --- | --- | --- | --- | --- | --- | --- | --- |
|  | |  | |  | | Mod. Ind. | | EPC | |
| qS11 |  | ↔ |  | qS12 |  | 192.139 |  | -0.493 |  |
| qS13 |  | ↔ |  | qS14 |  | 112.369 |  | -0.444 |  |
| qS9 |  | ↔ |  | qS14 |  | 37.112 |  | -0.287 |  |
| qS12 |  | ↔ |  | qS15 |  | 32.199 |  | -0.448 |  |
| qS5 |  | ↔ |  | qS15 |  | 29.011 |  | -0.245 |  |
| qS9 |  | ↔ |  | qS13 |  | 17.345 |  | -0.216 |  |
| qF4 |  | ↔ |  | qS14 |  | 17.129 |  | 0.275 |  |
| qF3 |  | ↔ |  | qS11 |  | 13.866 |  | 0.240 |  |
| qF6 |  | ↔ |  | qS14 |  | 12.555 |  | 0.232 |  |
| qS8 |  | ↔ |  | qS14 |  | 12.178 |  | -0.191 |  |
| qS8 |  | ↔ |  | qS9 |  | 11.426 |  | -0.188 |  |
|  | | | | | | | | | |

Due to their local dependency revealed by high residual covariance, we decided to remove items qS11 (insomnia), qS12 (sedatives) and qS14 (weakness), as well as qF4 (home mobility) and qF6 (supervision). We retained items qS13 (pain) and qS9 (depression) because they were considered essential for FLIGS-FQ.

Items qF7 (Accompaniment) and qS4 (Polypharmacy) were removed due to their differing functions in the two sexes, as revealed by the MH test and subsequent false discovery rate verification (see below).

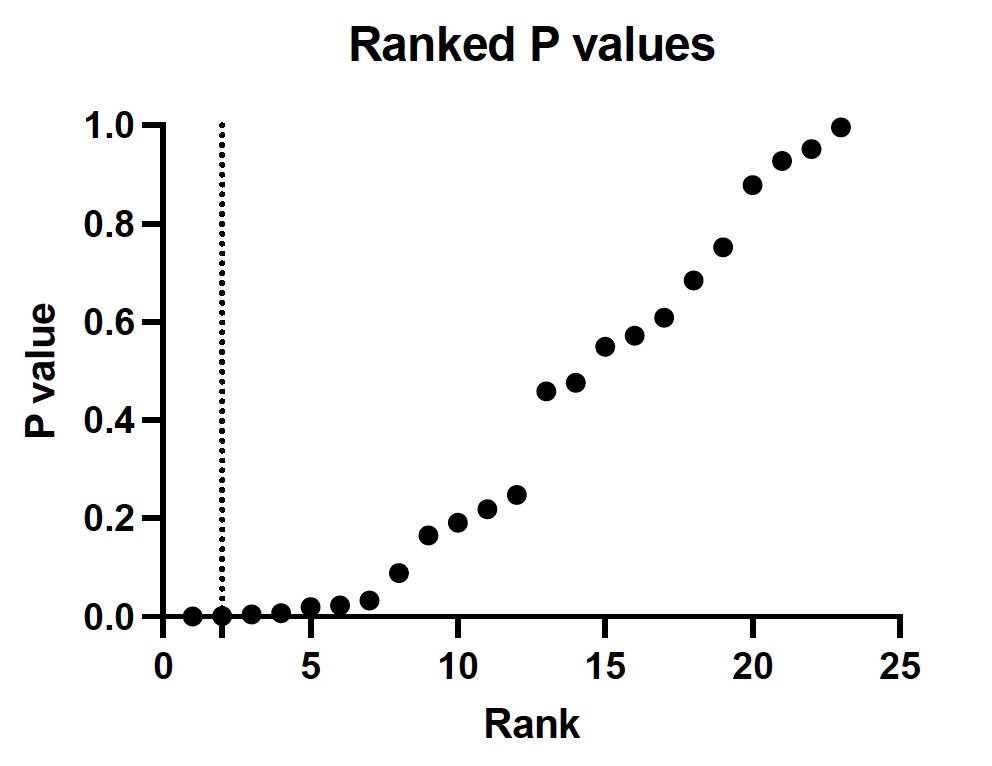


Method: Two-stage linear step-up procedure of Benjamini, Krieger and Yekutieli Q: 1%

Estimated number of true null hypotheses: 22. Threshold: P values less than 0.0009 are "discoveries"

**Confirmatory Factor Analysis (CFA) of FLIGS-FQ-16**

Estimator: WLSMV; Standard error calculation: robust.

| **Table 2. The items in FLIGS-FQ-16** | | |
| --- | --- | --- |
|  | | **Short Label** |
| **Functional Limitations** | |  |
| qF1 | Is supported by a caregiver or relative for more than 6 hours a day | Caregiver |
| qF2 | Must be followed or helped to bathe or shower | Bathing |
| qF3 | Needs help getting dressed | Dressing |
| qF5 | Needs help to manage medications | Medications |
| qF8 | Must use a cane or other aids to walk or move around outside the home | Mobility aids |
| **Geriatric Syndromes** | | |
| qS1 | Has frequent dizziness or balance problems | Balance |
| qS2 | Has serious vision problems | Poor vision |
| qS3 | Has serious hearing problems or use a hearing aid | Poor hearing |
| qS5 | Has major memory problems | Amnesia |
| qS6 | Has fallen in the last 6 months | Falls |
| qS7 | Has difficulty swallowing or often coughs when drinking | Dysphagia |
| qS8 | Lost a lot of weight in the past 6 months | Weight loss |
| qS9 | Often feels down or depressed | Depression |
| qS10 | Has incontinence problems and use pads to avoid getting wet | Incontinence |
| qS13 | Often complains of pain | Pain |
| qS15 | Has behavior problems | Behavior |

| *Chi-square test* | | | | | | | |
| --- | --- | --- | --- | --- | --- | --- | --- |
| Model | | Χ² | | df | | p | |
| Baseline model |  | 11710.352 |  | 120 |  |  |  |
| Factor model |  | 312.977 |  | 104 |  | < .001 |  |
|  | | | | | | | |

| *Fit indices* | | | |
| --- | --- | --- | --- |
| Index | | Value | |
| Comparative Fit Index (CFI) |  | 0.982 |  |
| Tucker-Lewis Index (TLI) |  | 0.979 |  |
| Bentler-Bonett Non-normed Fit Index (NNFI) |  | 0.979 |  |
| Bentler-Bonett Normed Fit Index (NFI) |  | 0.973 |  |
| Parsimony Normed Fit Index (PNFI) |  | 0.857 |  |
| Bollen's Relative Fit Index (RFI) |  | 0.969 |  |
| Bollen's Incremental Fit Index (IFI) |  | 0.982 |  |
| Relative Noncentrality Index (RNI) |  | 0.982 |  |
|  | | | |
| Note.  Except for the PNFI, the fit indices are scaled because of categorical variables in the data. | | | |

| *Other fit measures* | | | |
| --- | --- | --- | --- |
| Metric | | Value | |
| Root mean square error of approximation (RMSEA) |  | 0.047 |  |
| RMSEA 90% CI lower bound |  | 0.041 |  |
| RMSEA 90% CI upper bound |  | 0.053 |  |
| RMSEA p-value |  | 0.764 |  |
| Standardized root mean square residual (SRMR) |  | 0.075 |  |
| Hoelter's critical N (α = .05) |  | 515.503 |  |
| Hoelter's critical N (α = .01) |  | 562.059 |  |
| Goodness of fit index (GFI) |  | 0.991 |  |
| McDonald fit index (MFI) |  | 0.935 |  |
| Expected cross validation index (ECVI) |  |  |  |
|  | | | |
| Note.  The RMSEA results are scaled because of categorical variables in the data. | | | |

| *Average variance extracted* | | | |
| --- | --- | --- | --- |
| Factor | | AVE | |
| F |  | 0.513 |  |
|  | | | |

| *Reliability* | | | | | |
| --- | --- | --- | --- | --- | --- |
|  | | Coefficient ω | | Coefficient α | |
| F |  | 0.879 |  | 0.855 |  |
|  | | | | | |

**Parameter estimates**

| *Factor loadings* | | | | | | | | | | | | | | | |
| --- | --- | --- | --- | --- | --- | --- | --- | --- | --- | --- | --- | --- | --- | --- | --- |
|  | | | | | | | | | | | | 95% Confidence Interval | | | |
| Factor | | Indicator | | Estimate | | Std. Error | | z-value | | p | | Lower | | Upper | |
| F |  | qF1 |  | 0.911 |  | 0.016 |  | 58.340 |  | < .001 |  | 0.881 |  | 0.942 |  |
|  |  | qF2 |  | 0.971 |  | 0.009 |  | 102.248 |  | < .001 |  | 0.952 |  | 0.990 |  |
|  |  | qF3 |  | 0.899 |  | 0.017 |  | 51.473 |  | < .001 |  | 0.865 |  | 0.934 |  |
|  |  | qF5 |  | 0.912 |  | 0.016 |  | 57.922 |  | < .001 |  | 0.881 |  | 0.943 |  |
|  |  | qF8 |  | 0.820 |  | 0.023 |  | 35.046 |  | < .001 |  | 0.774 |  | 0.866 |  |
|  |  | qS1 |  | 0.794 |  | 0.027 |  | 29.891 |  | < .001 |  | 0.742 |  | 0.846 |  |
|  |  | qS2 |  | 0.486 |  | 0.050 |  | 9.642 |  | < .001 |  | 0.387 |  | 0.584 |  |
|  |  | qS3 |  | 0.438 |  | 0.049 |  | 8.880 |  | < .001 |  | 0.341 |  | 0.534 |  |
|  |  | qS5 |  | 0.814 |  | 0.027 |  | 29.777 |  | < .001 |  | 0.760 |  | 0.867 |  |
|  |  | qS6 |  | 0.520 |  | 0.043 |  | 12.210 |  | < .001 |  | 0.437 |  | 0.603 |  |
|  |  | qS7 |  | 0.665 |  | 0.041 |  | 16.407 |  | < .001 |  | 0.586 |  | 0.744 |  |
|  |  | qS8 |  | 0.463 |  | 0.046 |  | 10.156 |  | < .001 |  | 0.374 |  | 0.552 |  |
|  |  | qS9 |  | 0.487 |  | 0.043 |  | 11.376 |  | < .001 |  | 0.403 |  | 0.571 |  |
|  |  | qS10 |  | 0.716 |  | 0.031 |  | 22.998 |  | < .001 |  | 0.655 |  | 0.777 |  |
|  |  | qS13 |  | 0.374 |  | 0.046 |  | 8.217 |  | < .001 |  | 0.285 |  | 0.463 |  |
|  |  | qS15 |  | 0.765 |  | 0.046 |  | 16.540 |  | < .001 |  | 0.675 |  | 0.856 |  |
|  | | | | | | | | | | | | | | | |

| *Factor loadings* | | | | | | | | | | | | | | | |
| --- | --- | --- | --- | --- | --- | --- | --- | --- | --- | --- | --- | --- | --- | --- | --- |
|  | | | | | | | | | | | | 95% Confidence Interval | | | |
| Factor | | Indicator | | Std. estimate | | Std. Error | | z-value | | p | | Lower | | Upper | |
| F |  | qF1 |  | 0.911 |  | 0.016 |  | 58.340 |  | < .001 |  | 0.881 |  | 0.942 |  |
|  |  | qF2 |  | 0.971 |  | 0.009 |  | 102.248 |  | < .001 |  | 0.952 |  | 0.990 |  |
|  |  | qF3 |  | 0.899 |  | 0.017 |  | 51.473 |  | < .001 |  | 0.865 |  | 0.934 |  |
|  |  | qF5 |  | 0.912 |  | 0.016 |  | 57.922 |  | < .001 |  | 0.881 |  | 0.943 |  |
|  |  | qF8 |  | 0.820 |  | 0.023 |  | 35.046 |  | < .001 |  | 0.774 |  | 0.866 |  |
|  |  | qS1 |  | 0.794 |  | 0.027 |  | 29.891 |  | < .001 |  | 0.742 |  | 0.846 |  |
|  |  | qS2 |  | 0.486 |  | 0.050 |  | 9.642 |  | < .001 |  | 0.387 |  | 0.584 |  |
|  |  | qS3 |  | 0.438 |  | 0.049 |  | 8.880 |  | < .001 |  | 0.341 |  | 0.534 |  |
|  |  | qS5 |  | 0.814 |  | 0.027 |  | 29.777 |  | < .001 |  | 0.760 |  | 0.867 |  |
|  |  | qS6 |  | 0.520 |  | 0.043 |  | 12.210 |  | < .001 |  | 0.437 |  | 0.603 |  |
|  |  | qS7 |  | 0.665 |  | 0.041 |  | 16.407 |  | < .001 |  | 0.586 |  | 0.744 |  |
|  |  | qS8 |  | 0.463 |  | 0.046 |  | 10.156 |  | < .001 |  | 0.374 |  | 0.552 |  |
|  |  | qS9 |  | 0.487 |  | 0.043 |  | 11.376 |  | < .001 |  | 0.403 |  | 0.571 |  |
|  |  | qS10 |  | 0.716 |  | 0.031 |  | 22.998 |  | < .001 |  | 0.655 |  | 0.777 |  |
|  |  | qS13 |  | 0.374 |  | 0.046 |  | 8.217 |  | < .001 |  | 0.285 |  | 0.463 |  |
|  |  | qS15 |  | 0.765 |  | 0.046 |  | 16.540 |  | < .001 |  | 0.675 |  | 0.856 |  |
|  | | | | | | | | | | | | | | | |

### Modification Indices

| *Residual covariances* | | | | | | | | | |
| --- | --- | --- | --- | --- | --- | --- | --- | --- | --- |
|  | |  | |  | | Mod. Ind. | | EPC | |
| qS5 |  | ↔ |  | qS15 |  | 26.302 |  | -0.247 |  |
| qS9 |  | ↔ |  | qS13 |  | 21.342 |  | -0.241 |  |
| qS8 |  | ↔ |  | qS9 |  | 12.601 |  | -0.199 |  |
| qF2 |  | ↔ |  | qF3 |  | 10.307 |  | -0.090 |  |
|  | | | | | | | | | |

**Item Response Theory analysis**

The table shows the estimates obtained by running the one-parameter logistic (Rash) model with clustered bootstrap resampling by group membership and gender.

The items share a common discrimination and are ordered from hardest to easiest.
